# Pernicious Swindles

**Published:** 1889-09

**Authors:** 


### PERNICIOUS SWINDLES.

When the "limb of satan" who invented the lie of *Scotch Oats Essence*, to make drunkards of unsuspecting people by trick and device, was suppressed by exposure in that enterprise, we predicted that he would soon turn up again, in some similar phase of diabolical mischief. That was but a few weeks since, and already he is reported on the market once more, at the old stand, too, with a cure for the opium habit, which means a way to gratify the opium craving and complete the ruin of the victim under a false pretense with enormous factitious profits to the vender of the disguised drug or its analogues.

To the best of our information and belief—and we have taken some pains in experimenting—the advertised opium cures, so far as they are not opium itself in disguise, generally consists of substitutes that take its place only to complete its work with ugly variations of their own, such as cocoa, hasheesh, and various other narcotics are able to contribute. It is high time that this business was taken in hand by the authorities. It is a monstrous state of lawlessness, when secret preparations of drugs can be freely put on sale and pushed into use as common as food, by any ignoramus or scoundrel who chooses to make his money in that way. We make it a misdemeanor to prescribe for a few individuals, privately, without the credentials of professional science and honor ; but the wholesale prescriber is a "chartered libertine," responsible neither to medical, excise, or public health laws, for anything he may do. Every medicinal preparation allowed to be sold should be first approved as legitimate by the medical authorities, and its exact composition ascertained and stated on all its labels. If such publicity were to destroy the popular market for nostrums there would be none to mourn but the nostrum-makers.—*Sanitary Era.*

